# Trophic and Non-Trophic Interactions in a Biodiversity Experiment Assessed by Next-Generation Sequencing

**DOI:** 10.1371/journal.pone.0148781

**Published:** 2016-02-09

**Authors:** Julia Tiede, Bernd Wemheuer, Michael Traugott, Rolf Daniel, Teja Tscharntke, Anne Ebeling, Christoph Scherber

**Affiliations:** 1 Agroecology, Department of Crop Sciences, Georg-August University Goettingen, Grisebachstr. 6, 37077, Goettingen, Germany; 2 Institute of Microbiology and Genetics, Department of Genomic and Applied Microbiology, Georg-August University Goettingen, Grisebachstr. 8, 37077, Goettingen, Germany; 3 Mountain Agriculture Research Unit, Institute of Ecology, University of Innsbruck, Technikerstrasse 25, 6020, Innsbruck, Austria; 4 Institute of Ecology, Friedrich-Schiller-University Jena, Dornburger Str. 159, 07743, Jena, Germany; 5 Institute of Landscape Ecology, University of Muenster, Heisenbergstr. 2, 48149, Muenster, Germany; Oklahoma State University, UNITED STATES

## Abstract

Plant diversity affects species richness and abundance of taxa at higher trophic levels. However, plant diversity effects on omnivores (feeding on multiple trophic levels) and their trophic and non-trophic interactions are not yet studied because appropriate methods were lacking. A promising approach is the DNA-based analysis of gut contents using next generation sequencing (NGS) technologies. Here, we integrate NGS-based analysis into the framework of a biodiversity experiment where plant taxonomic and functional diversity were manipulated to directly assess environmental interactions involving the omnivorous ground beetle *Pterostichus melanarius*. Beetle regurgitates were used for NGS-based analysis with universal 18S rDNA primers for eukaryotes. We detected a wide range of taxa with the NGS approach in regurgitates, including organisms representing trophic, phoretic, parasitic, and neutral interactions with *P*. *melanarius*. Our findings suggest that the frequency of (i) trophic interactions increased with plant diversity and vegetation cover; (ii) intraguild predation increased with vegetation cover, and (iii) neutral interactions with organisms such as fungi and protists increased with vegetation cover. Experimentally manipulated plant diversity likely affects multitrophic interactions involving omnivorous consumers. Our study therefore shows that trophic and non-trophic interactions can be assessed via NGS to address fundamental questions in biodiversity research.

## Introduction

Biodiversity in terrestrial ecosystems is declining due to intensified land use and other human-driven environmental changes [1–3]. How such a decline in diversity affects ecosystem functioning is studied most often for plant diversity loss, including both natural systems [4] and controlled experiments with manipulated plant communities (e.g. [2]). For decades, plant diversity experiments have focused on productivity [2,5], while more recent research investigates how the diversity of primary producers affects higher trophic levels [6,7]. These studies show that plant species richness has cascading, bottom-up effects on abundance and species richness of higher trophic levels [8–11]. However, the assignment of organisms to trophic groups (such as herbivores, carnivores, or omnivores) is so far mostly based on literature data [12], combined with information on morphology and ecology [13]. In addition, it is difficult to relate organism abundances to process rates such as herbivory or predation, because a species may not consume food proportional to its abundance [14–16]. A further complication arises if consumers are omnivores that feed at more than one trophic level. While omnivores are abundant in many systems [17], their responses to plant diversity remain elusive.

A promising approach to directly assess trophic interactions is the DNA-based detection of food remains in gut contents, which is widely used to study trophic interactions in various ecosystems [18–22]. Sequence-based identification of food DNA using next generation sequencing (NGS), combined with universal primers for common barcoding regions, allows simultaneous detection of feeding events from a wide range of potential interaction partners [23–25]. In addition to food items, NGS-based methods often co-sequence DNA of other organisms encountered in the environment [18]. Information on interactions involving these organisms is usually discarded in dietary studies [26, 27], but may indicate non-trophic interactions, such as commensalism or neutralism that are often completely ignored in ecological networks [28]. This approach, albeit ideally suited to empirically assess interactions in biodiverse communities, has not yet been applied to study the effects of plant diversity on trophic and non-trophic processes.

Here, we use the framework of a grassland biodiversity experiment to test the potential of NGS for the direct and simultaneous assessment of trophic and non-trophic interactions and analyze how these interactions are affected by plant biodiversity. We use the omnivorous ground beetle *Pterostichus melanarius* Illiger (Coleoptera; Carabidae) as a model species, as it is geographically widespread, locally abundant and present in many natural and agricultural ecosystems. *Pterostichus melanarius* primarily feeds on a wide range of invertebrates from various trophic levels but its diet also includes plant material [29–31]. Furthermore, *P*. *melanarius* regurgitates its gut content in response to mechanical or thermal stress, allowing non-invasive and non-lethal collection of gut contents [32]. Another advantage of using regurgitates instead of whole body DNA extracts of beetles is that they may be ideally suited for sequence-based identification of ingested organisms using universal primers without the need to include blocking primers because only little DNA of the consumer should be present in this sample type [33]. Blocking primers are the most commonly used approach to overcome the problem that universal primers, which also amplify consumer DNA, primarily generate amplicons of the consumer that limit the detection of less abundant and/or highly digested DNA of food remains [34]. Blocking primers are consumer-specific oligonucleotides that inhibit the amplification of specific DNA sequences [35]. In addition to consumer DNA, however, blocking primers can co-block related non-target species [36] and testing the specificity of blocking primers is often impractical in field studies with many, also unknown, prey species. An alternative approach is to compensate for consumer co-amplification by increasing sequencing depth [36,37]. However, if regurgitates are used, blocking primers might not be necessary because regurgitates may contain much less consumer DNA. Regurgitates of invertebrates are successfully used in combination with prey-specific primers [32,33] but their potential for NGS-based diet analysis with universal primers is not yettested.

The aim of this study is to assess the potential of NGS-based gut content-analysis to study multitrophic interactions in response to changes in biodiversity. Within the framework of a plant diversity experiment, we test if regurgitates of an abundant omnivore can be analysed with NGS by applying universal primers without blocking primers. By simultaneously analysing trophic and non-trophic interactions, we exploit the full potential of NGS to assess the impact of biodiversity on interspecific interactions.

## Material and Methods

### Ethics statement

Arthropod sampling was conducted with the permission of the city council of Jena, Germany.

### Study site

**Fig 1:**
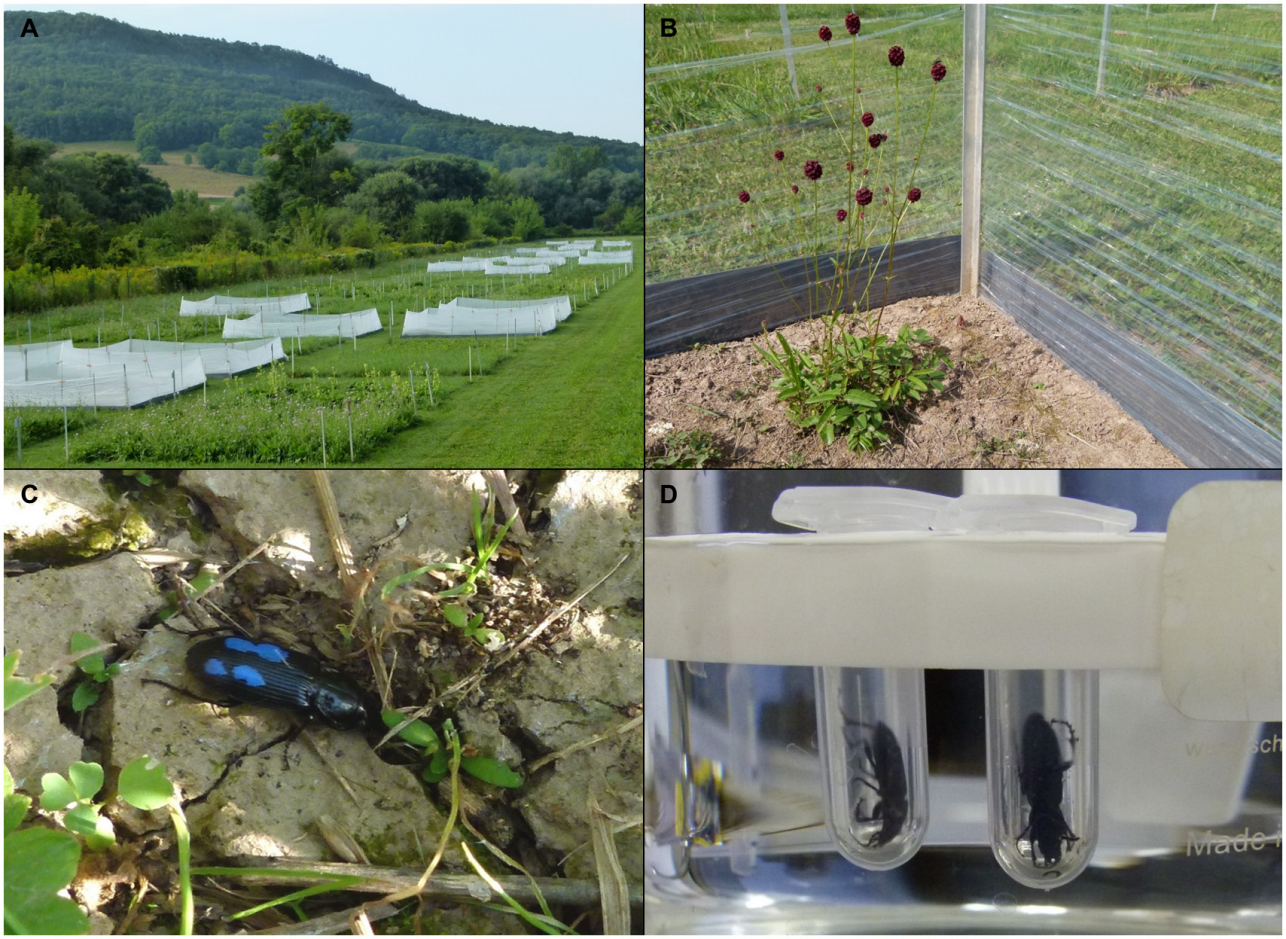
Setup of field experiment and regurgitate sampling. (A) Overview of plots of the Trait-Based Experiment with enclosures. (B) Enclosures were made of construction foil sunk into the soil using PVC panels. (C) Marked beetles were released and recaptured to sample regurgitates (D) sampling regurgitates. Photographs by J. Tiede.

This study was conducted within the framework of a grassland biodiversity experiment (The Jena Experiment; Thuringia, Germany, 50°95′ N, 11°63′ E, 130 m above sea level) [38] in experimental plots of the Trait-Based Diversity Experiment (TBE; [39]). The species pool in the TBE consists of 20 Central European grass and non-legume herbaceous species. Plant communities were manipulated to cover a gradient of plant species richness (1, 2, 3, 4, and 8) and plant functional diversity (1, 2, 3, and 4) on 138 plots (3.5 m x 3.5 m). The gradient of plant functional diversity was based on plant traits known to be important for spatial and temporal resource use such as plant height, rooting depth, or phenology, and represents the levels from low (1) to high (4) trait complementarity in the plant community [39]. The experimental plots were maintained by biannual mowing and weeded three times per year to remove unwanted species. In addition to the experimentally manipulated variables (plant species richness and plant functional diversity), we visually estimated vegetation cover (in percent) in mid-August 2013. For logistic reasons only a subset of the 138 plots was used for this study. 33 plots were selected at random: including 10 monocultures, five two-species mixtures, five three-species mixtures, ten four-species mixtures, and all three eight-species mixtures. Thus, our sampling design had more replicates at low (1) and high (4,8) plant species richness, which minimizes the standard error of the slope in subsequent statistical analyses [40]. Plant biomass data from the previous year was used to show that the 33 plots selected did not introduce a systematic bias compared to the full 138 plots. Every plot was fenced with an enclosure for a period of two weeks in August 2013 to prevent inter-plot movement of *P*. *melanarius* and other ground-dwelling organisms. For the enclosures, transparent construction foil (PE, 20 μ, Rajapack, Ettlingen) was wrapped around the four corner poles of each plot (~50 cm height) and sunk into the soil using PVC panels (~15 cm depth) (Fig 1A and 1B).

### Study organism

Adult *P*. *melanarius* were collected in the weeks preceding the experiment using dry pitfall traps at different locations around Göttingen (Germany; 51°55′ N, 9°95′ E) in July 2013 as well as in the surrounding grass margins of the Jena Experiment in August 2013. Beetles were kept in plastic containers on a substrate of moist clay pebbles in a dark room at 18°C and maintained on cat food (K-Classic Adult, Kaufland AG, Germany) but starved 48 h before the experiment. On August 15, five beetles were released per plot; each beetle was marked with an individual pattern of coloured dots on its elytra (Fig 1C). After allowing the released beetles to acclimatise to the plot conditions for four days, we repeatedly recaptured them over a period of 10 days in one central dry pitfall trap (4.5 cm diameter). The traps were filled with clay pebbles and emptied in the morning and evening to minimize within-trap predation events. If remains of other organisms were found in a trap, all beetles caught in this trap were excluded from analyses. To sample the beetles’ gut contents, we placed them individually headfirst in 1.5 mL reaction tubes and exposed the tubes for a few seconds to hot water (~60°C) to induce regurgitation (Fig 1D). Regurgitates were immediately frozen at -18°C and subsequently stored at -80°C. Afterwards, the beetles were released on the original plot. We were not able to recapture beetles from all plots because only one trap per plot was used for a comparatively short recapture period of 10 days, due to other experiments conducted on the same plots. Additionally, some of the beetles failed to regurgitate or the amount of stomach content was too low for analysis. Several other samples dropped out during the analysis process, so that our final dataset represented 13 plots.

### DNA extraction

Total DNA was extracted from regurgitates in a molecular diagnostic laboratory at the Institute of Ecology, University of Innsbruck, Austria. Each regurgitate sample was mixed with 200 μL lysis buffer containing 5 μL Proteinase K (10 mg/mL, AppliChem, Darmstadt, Germany) and TES-buffer (0.1 M TRIS, 10 mM EDTA, 2% SDS, pH 8) and was incubated at 56°C for 3 h. The DNA was extracted from the lysate on a BioSprint 96 robotic DNA extraction platform using the MagAttract DNA Blood M96 Kit (Qiagen, Hilden, Germany). Four negative extraction controls (DNA extraction blanks) were included to monitor for carry-over DNA contamination during the extraction process and were subsequently tested in PCR reactions for NGS.

### Next generation sequencing and sequence processing

Next generation sequencing of regurgitates was conducted at the Department of Genomic and Applied Microbiology (University of Goettingen, Germany). To analyse a broad spectrum of ingested organisms from the regurgitates of *P*. *melanarius* without *a priori* decisions on focal groups, we used universal primers amplifying a ~600 bp region of the eukaryotic 18S rDNA gene: F515 (5’GTGCCAGCMGCCGCGGTAA-3’) and R1119 (5’-GGTGCCCTTCCGTCA-3’) [41]. Taxon coverage of the primer pair was previously tested *in silico* using Primer Prospector [42] and reference sequences derived from the SILVA database [43]. The primers included a Roche 454 pyrosequencing adaptor, a library key sequence, and a multiplex identifier (MID). Each 50 μL PCR reaction contained 10 μL of 5x Phusion GC buffer (Finnzymes, Vantaa, Finland), 0.2mM of each dNTP, 4 μM of each primer, 1.5 μL dimethyl sulfoxide (DMSO), 1 U Phusion Hot Start DNA polymerase (Finnzymes), 1 μL template DNA, and 32 μL diethylpyrocarbonate (DEPC) water. The thermocycling protocol was 98°C for 30 s, 35 cycles of 98°C for 10 s, 60°C for 20 s, 72°C for 20 s, and 72°C for 5 min once. One template-free control was included in every PCR run. Samples that showed PCR products on agarose gel were amplified in three technical replicates, purified with the peqGOLD Gel Extraction Kit (Peqlab, Erlangen, Germany) and pooled at equal DNA concentrations. DNA concentration was quantified using a Qubit fluorometer (Invitrogen, Carlsbad, USA) with the Quant-iT dsDNA HS assay kit; 20 regurgitates with a sufficient DNA concentration (≥ 2 ng μl^-1^) were sequenced.

The sequencing was carried out on a GS-FLX+ 454 pyrosequencer using Titanium chemistry (Roche, Branford, CT), with a targeted surveying effort of 5,000 reads per sample. Short reads (<200 bp), and low quality reads (homopolymer stretches >8 bp; primer mismatches >5 bp) were removed using QIIME v1.6 [44]. The sequences were denoised using Acacia v1.52 [45] and cutadapt was used to truncate remaining primer sequences [46]. Chimeric sequences were removed using UCHIME [47] in reference mode with SILVA (SSURef 119 NR database as reference data set [43]). Using the UCLUST algorithm [48], the remaining sequences were clustered in operational taxonomic units (OTUs) at 99% genetic similarity. The consensus sequences were calculated using USEARCH (v. 7.0.1090). OTUs were subsequently classified by blast alignment against the SILVA database [49]. The taxonomy of the best hit was assigned to the respective OTU. DNA sequences were deposited in the Sequence Read Archive (SRA) of the National Center for Biotechnology Information under accession SRA282133.

### Data processing

Two samples were excluded because of low numbers of total sequences or high numbers of consumer (*P*. *melanarius*) sequences. For the analysis of taxa composition in the remaining 18 regurgitate samples, we removed all OTUs classified as consumer (1 OTU, 1 sample), human (1 OTU, 7 samples), vertebrate (1OTU, 1 sample), tree species (5 OTU, 1–5 samples) and aquatic species (6 OTU, 1–2 samples). DNA of aquatic species might have originated from a flooding event in June 2013 [50], and tree DNA likely originated from pollen of trees growing nearby in northern and eastern direction. Human and vertebrate DNA (squirrel) likely represented contaminations. In addition, we excluded OTUs that could not be classified to order-level (4 OTUs, 1–4 samples), singletons and doubletons (46 OTU, 1–4 samples) from the analysis. A complete list of removed OTUs is provided in S1 Table.

For the analysis of interaction types, all remaining OTUs were aggregated at genus level and grouped based on literature information on their most likely interaction with *P*. *melanarius* (Table 1). We differentiated between trophic interactions that are beneficial (+) to *P*. *melanarius* but negative (-) for the interaction partner, and non-trophic interactions that are neutral (0) or negative for the beetle and beneficial or neutral for the interaction partner.

**Table 1:** Organisms detected with NGS in regurgitates of *P*. *melanarius*, sorted by their most likely type of interaction with the beetle.

Interaction type	Kingdom	Phylum	Class	Order	Family	Genus	Nutrition, metabolism	N
**Trophic (feeding, +/-)**	Plantae	Tracheophyta	Magnoliopsida	Asterales	Asteraceae	*Artemisia*	autotrophic	1
	Plantae	Tracheophyta	Magnoliopsida	Caryophyllales	Polygonaceae	*Rumex*	autotrophic	4
	Plantae	Tracheophyta	Magnoliopsida	Dipsacales	Caprifoliaceae	*Triplostegia*	autotrophic	3
	Plantae	Tracheophyta	Magnoliopsida	Fabales	Fabaceae	n/a	autotrophic	3
	Plantae	Tracheophyta	Magnoliopsida	Gentianales	Rubiaceae*	Guettarda*	autotrophic	1
	Plantae	Tracheophyta	Magnoliopsida	Lamiales	Plantaginaceae	*Plantago*	autotrophic	2
	Plantae	Tracheophyta	Magnoliopsida	Poales	Poaceae	*Triticum*	autotrophic	3
	Plantae	Tracheophyta	Magnoliopsida	Poales	Restionaceae	n/a	autotrophic	1
	Plantae	Tracheophyta	Magnoliopsida	Ranunculales	Ranunculaceae	*Ranunculus*	autotrophic	1
	Plantae	Tracheophyta	Magnoliopsida	Rosales	Rosaceae	*Prunus*	autotrophic	3
	Plantae	Tracheophyta	Magnoliopsida	Rosales	Urticaceae	*Urtica*	autotrophic	8
	Plantae	Tracheophyta	Magnoliopsida	Rosales	n/a	n/a	autotrophic	4
	Animalia	Annelida	Clitellata	Haplotaxida	Hormogastridae*	Hormogaster*	detrivorous	1
	Animalia	Arthropoda	Arachnida	Araneae	Salticidae	Goleba*	predatory	1
	Animalia	Arthropoda	Arachnida	Araneae	Sparassidae	*Micrommata*^+^	predatory	1
	Animalia	Arthropoda	Arachnida	Sarcoptiformes	Glycyphagidae	Alabidopus*	fungivorous	1
	Animalia	Arthropoda	Arachnida	Sarcoptiformes	Oribatulidae	*Oribatula*	detrivorous	1
	Animalia	Arthropoda	Arachnida	Trombidiformes	Microtrombidiidae	*Microtrombidium*	parasitic on vertebrates	1
	Animalia	Arthropoda	Arachnida	Trombidiformes	Trombiculidae	n/a	predatory	3
	Animalia	Arthropoda	Insecta	Coleoptera	Carabidae	*Bembidion*	predatory	4
	Animalia	Arthropoda	Insecta	Dermaptera	Forficulidae	n/a	detrivorous	1
	Animalia	Arthropoda	Insecta	Orthoptera	Acrididae	*Gomphocerus*	herbivorous	1
	Animalia	Mollusca	Gastropoda	Stylommatophora	Agriolimacidae	*Deroceras*	herbivorous	2
	Animalia	Mollusca	Gastropoda	Stylommatophora	Hygromiidae	*Helicella*	herbivorous	1
**Parasitism (-/+)**	Chromista	Miozoa	Conoidasida	Eugregarinorida	n/a	n/a	parasitic on insects	2
	Fungi	Ascomycota	Sordariomycetes	Hypocreales	Cordycipitaceae	*Isaria*	entomopathogenic	3
**Phoresy (0/+)**	Animalia	Arthropoda	Arachnida	Sarcoptiformes	Acaridae	*Histiogaster*	bacterivorous	3
	Animalia	Arthropoda	Arachnida	Sarcoptiformes	Histiostomatidae	*Anoetus*	bacterivorous	1
	Animalia	Arthropoda	Arachnida	Sarcoptiformes	Histiostomatidae	n/a	bacterivorous	7
**Neutralism (0/0)**	Chromista	Cercozoa	Gromiidea	Reticulosida	Gymnophryidae	*Gymnophrys*	omnivorous	2
	Chromista	Cercozoa	Sarcomonadea	Cercomonadida	Heteromitidae	*Heteromita*	bacterivorous	4
	Chromista	Cercozoa	Sarcomonadea	Cercomonadida	n/a	*Cercomonas*	bacterivorous	3
	Chromista	Cercozoa	Sarcomonadea	Glissomonadida	Bodomorphidae	*Bodomorpha*	bacterivorous	1
	Chromista	Cercozoa	Sarcomonadea	Glissomonadida	n/a	n/a	bacterivorous	1
	Chromista	Cercozoa	Thecofilosa	Cryomonadida	Rhizaspididae	*Rhogostoma*	bacterivorous	3
	Chromista	Cercozoa	Vampyrellidea	Vampyrellida	Vampyrellidae	n/a	omnivorous	1
	Chromista	Ciliophora	Colpodea	Colpodida	Colpodidae	*Exocolpoda*	bacterivorous	1
	Chromista	Miozoa	Apicomonadea	Colpodellida	Colpodellidae	*Colpodella*	predatory on protists	1
	Chromista	Pseudofungi	Hyphochytrea	Hyphochytriida	n/a	n/a	phytopathogenic	1
	Chromista	Pseudofungi	Oomycetes	Pythiales	Pythiaceae	*Pythium*	phytopathogenic	1
	Fungi	Ascomycota	Dothideomycetes	Acrospermales	Acrospermaceae	*Acrospermum*	saprotrophic	1
	Fungi	Ascomycota	Dothideomycetes	Capnodiales	n/a	n/a	phytopathogenic	11
	Fungi	Ascomycota	Dothideomycetes	Pleosporales	Didymellaceae	*Didymella*	phytopathogenic	1
	Fungi	Ascomycota	Dothideomycetes	Pleosporales	Didymellaceae	*Phoma*	phytopathogenic	8
	Fungi	Ascomycota	Dothideomycetes	Pleosporales	Phaeosphaeriaceae	*Parastagonospora*	phytopathogenic	3
	Fungi	Ascomycota	Dothideomycetes	Pleosporales	Pleosporaceae	*Pyrenophora*	phytopathogenic	1
	Fungi	Ascomycota	Dothideomycetes	Pleosporales	Tubeufiaceae	*Tubeufia*	saprotrophic	2
	Fungi	Ascomycota	Dothideomycetes	Pleosporales	n/a	n/a	saprotrophic	9
	Fungi	Ascomycota	Eurotiomycetes	Chaetothyriomycetidae	Herpotrichiellaceae	*Coniosporium*	n/a	1
	Fungi	Ascomycota	Eurotiomycetes	Eurotiales	Trichocomaceae	*Penicillium*	saprotrophic	1
	Fungi	Ascomycota	Leotiomycetes	Helotiales	Helotiaceae	*Cudoniella*	saprotrophic	3
	Fungi	Ascomycota	Leotiomycetes	Helotiales	Vibrisseaceae	*Phialocephala*	endophytic	1
	Fungi	Ascomycota	Leotiomycetes	Helotiales	n/a	n/a	n/a	2
	Fungi	Ascomycota	Pezizomycetes	Pezizales	n/a	n/a	saprotrophic	1
	Fungi	Ascomycota	Saccharomycetales	Saccharomycetales	Debaryomycetaceae	*Priceomyces*	n/a	5
	Fungi	Ascomycota	Saccharomycetales	Saccharomycetales	Dipodascaceae	*Yarrowia*	n/a	13
	Fungi	Ascomycota	Saccharomycetales	Saccharomycetales	Hanseniaspora	n/a	n/a	1
	Fungi	Ascomycota	Saccharomycetales	Saccharomycetales	Saccharomycetaceae	*Candida*	n/a	1
	Fungi	Ascomycota	Sordariomycetes	Hypocreales	Fusarium	*Fusarium*	phytopathogenic	2
	Fungi	Ascomycota	Sordariomycetes	Hypocreales	Hypocreaceae	*Acremonium*	phytopathogenic	1
	Fungi	Ascomycota	Sordariomycetes	Hypocreales	n/a	n/a	n/a	5
	Fungi	Ascomycota	Sordariomycetes	Sordariales	Chaetosphaeriaceae	*Chaetosphaeria*	phytopathogenic	1
	Fungi	Ascomycota	Sordariomycetes	Sordariales	Sordariaceae	*Neurospora*	saprotrophic	1
	Fungi	Ascomycota	Sordariomycetes	Xylariales	Hyponectriaceae	*Microdochium*	phytopathogenic	2
	Fungi	Basidiomycota	Agaricomycetes	Agaricales	Bolbitiaceae	*Conocybe*	saprotrophic	1
	Fungi	Basidiomycota	Agaricomycetes	Agaricales	Marasmiaceae	*Baeospora*	saprotrophic	1
	Fungi	Basidiomycota	Agaricomycetes	Agaricales	Physalacriaceae	*Hymenopellis*	saprotrophic	1
	Fungi	Basidiomycota	Agaricomycetes	Agaricales	Tricholomataceae	*Clitocybula*	saprotrophic	1
	Fungi	Basidiomycota	Agaricomycetes	Boletales	Hygrophoropsidaceae	*Leucogyrophana*	saprotrophic	1
	Fungi	Basidiomycota	Agaricomycetes	Hymenochaetales	Tubulicrinaceae	*Hyphodontia*	saprotrophic	1
	Fungi	Basidiomycota	Agaricomycetes	Polyporales	Polyporaceae	*Tyromyces*	saprotrophic	2
	Fungi	Basidiomycota	Exobasidiomycetes	n/a	n/a	*Tilletiopsis*	phytopathogenic	3
	Fungi	Basidiomycota	Microbotryomycetes	Heterogastridiales	Heterogastridiaceae	*Colacogloea*	saprotrophic	3
	Fungi	Basidiomycota	Microbotryomycetes	Heterogastridiales	Heterogastridiaceae	*Heterogastridium*	saprotrophic	11
	Fungi	Basidiomycota	Microbotryomycetes	Sporidiobolales	Sporidiobolaceae	*Rhodotorula*	saprotrophic	3
	Fungi	Basidiomycota	Microbotryomycetes	Sporidiobolales	Sporidiobolaceae	*Sporobolomyces*	saprotrophic	5
	Fungi	Basidiomycota	Microbotryomycetes	Sporidiobolales	n/a	n/a	n/a	1
	Fungi	Basidiomycota	n/a	Malasseziales	Malasseziaceae	*Malassezia*	animal-pathogenic	4
	Fungi	Basidiomycota	Pucciniomycetes	Pucciniales	Malasseziaceae	n/a	phytopathogenic	3
	Fungi	Basidiomycota	Tremellomycetes	Filobasidiales	Filobasidiaceae	n/a	n/a	1
	Fungi	Basidiomycota	Tremellomycetes	Tremellales	Tremellaceae	*Cryptococcus*	animal-pathogenic	3
	Fungi	Basidiomycota	Tremellomycetes	Tremellales	Tremellaceae	*Dioszegia*	parasitic on fungi	1
	Fungi	Basidiomycota	Tremellomycetes	Tremellales	n/a	n/a	n/a	4
	Fungi	Zygomycota	n/a	Mortierellales	n/a	n/a	saprotrophic	3
	Fungi	Zygomycota	n/a	Mucorales	Mucoraceae	*Mucor*	saprotrophic	4
	Plantae	Chlorophyta	Chlorophyceae	Chlamydomonadales	Dunaliellaceae	n/a	autotrophic	3
	Plantae	Chlorophyta	Chlorophyceae	Chlamydomonadales	Haematococcaceae	n/a	autotrophic	3
	Plantae	Chlorophyta	Trebouxiophyceae	Prasiolales	Prasiolaceae	*Stichococcus*	autotrophic	2
	Protozoa	Amoebozoa	Flabellinia	Vanellida	Vanellidae	*Vanella*	bacterivorous	1
	Protozoa	Amoebozoa	Myxogastrea	Physarida	Physaridae	*Physarum*	saprotrophic	1

Nutrition and metabolism indicate the most common source of energy uptake for the taxa, with predators and omnivores referred to as intraguild predation. “N” indicates the detection frequency. Taxonomy follows a Linnaean classification as proposed by [53].

* The closest match in the SILVA database is not endemic in Thuringia, Germany. In this case we consider the next higher taxonomic level as representative.

^+^ Since the spider family Sparassidae is represented only by the genus *Micrommata* in the sampling region, we added this information to the list of taxa.

Trophic interactions (+/-):
  - Total feeding interactions: all organisms that were likely actively consumed by *P*. *melanarius*
    - Plant derived food: higher plant taxa
    - Prey: all animal taxa except phoretic mites
      - Intraguild predation: prey with predatory or omnivorous nutrition
Non-trophic interactions:
  - Parasitism (-/+): organisms that presumably parasitize *P*. *melanarius*
  - Phoresy (0/+): mites that use insects as phoretic carriers and whose DNA could either originate from mites or mite remains that have fallen off during sampling
  - Neutralism (0/0): organisms without known interaction with *P*. *melanarius* that were likely passively consumed together with food

For the analysis of plant diversity effects on taxa detection in regurgitates, the number of OTUs in each group was calculated for each sample (S1 R-Script, S1 and S2 Data). Four plots were represented by two or three samples. For these, the number of taxa and the number of sequences per group were averaged and rounded to the smallest following integer (ceiling function). The resulting 13 independent data points represented 13 plots, including three monocultures, two two-species mixtures, three three-species mixtures, three four-species mixtures, and two eight-species mixtures.

### Statistical analysis

Data were analysed using R (version 3.1.2, R Development Core Team, 2014). We used generalized linear models (GLM) with negative binomial or quasipoisson errors to analyse the effects of the explanatory variables on the richness of OTUs for each group. Models included either plant species richness, functional diversity, or vegetation cover as explanatory variables, as these variables were colinear when entered together in single models; this resulted in a total of three individual models per OTU group. To account for potential effects of the number of sequences per OTU, we additionally ran quasipoisson models with number of sequences per OTU as known prior weights, giving more weight to samples with a high number of sequences. Note that the number of sequences cannot be used as a measure of consumed biomass as it is affected by the time since consumption and characteristics of the prey tissue that affect digestion time [24,36,51,52].

## Results

With NGS, we found a total of 90 OTUs in regurgitates of *P*. *melanarius*, covering a range of five kingdoms within the Eukaryotes [53]: Animalia, Chromista, Fungi, Plantae, and Protozoa. 77 OTUs were assigned to family level, covering 73 different families, and 67 to genus level, covering 63 different genera (Table 1).

### Detection of trophic and non-trophic interactions with NGS

Of these 90 OTUs, 24 were categorized as feeding interactions, comprising 12 plant and 12 animal taxa. Four of the identified plant taxa were locally present as part of the Trait-Based Experiment: the genera *Plantago* (Lamiales), *Ranunculus* (Ranunculales), and *Rumex* (Caryophyllales), and the family Poaceae (Poales). Other plant taxa, such as the stinging nettle *Urtica* (Rosales), were locally present in the vegetation matrix surrounding the plots and were occasional weeds in the experimental plots.

Animal prey detected using NGS included herbivores and detritivores, such as gastropods (Stylommatophora: *Deroceras*, and *Xerolenta*), mites (Trombidiformes: Microtrombidium; Sarcoptiformes: Glcyyphagidae, and *Orbitulata*), grasshoppers (Orthoptera: Gomphocerus), and earthworms (Haplotaxida: Hormogastridae). In addition, we detected other predator taxa: DNA of another ground beetle (Coleoptera: *Bembidion*) was found in four plots, a predatory mite (Trombidiformes: Trombiculidae) in three plots, an earwig (Dermaptera: Forficulidae), and two spider taxa (jumping spiders; Araneae: Salticidae, and a huntsman spider; Sparassidae, likely *Micrommata virescens*).

In addition to feeding interactions, we detected organisms that likely interacted negatively (parasites) or neutrally (commensalism, neutralism) with *P*.*melanarius* (Table 1). Two organisms that were presumably parasites of *P*. *melanarius* were present in samples from five plots: an entomopathogenic fungus (Ascomycota: Hypocreales: *Isaria* sp.) known to infect carabid beetles [54], and a group of parasitic protists (Apicomplexa: Eugregarinida) that frequently infects *P*. *melanarius* [55]. DNA of phoretic mites was found in regurgitates from 11 plots, with the family Histiostomatidae (Acariformes) represented eight times and the family Acaridae, genus *Histiogaster* sp. (Acariformes), found three times. None of the plots contained both families together. Most OTUs (N = 61) detected in the regurgitates of *P*. *melanarius* represented neutral interaction partners with no specific relation to the beetle (passive consumption, environmental DNA). Most of these organisms were fungi (N = 45), and protists (Amoebozoa and SAR, N = 13), but we also detected terrestrial algae (N = 3).

### Effects of plant biodiversity and vegetation cover on species interactions

**Fig 2:**
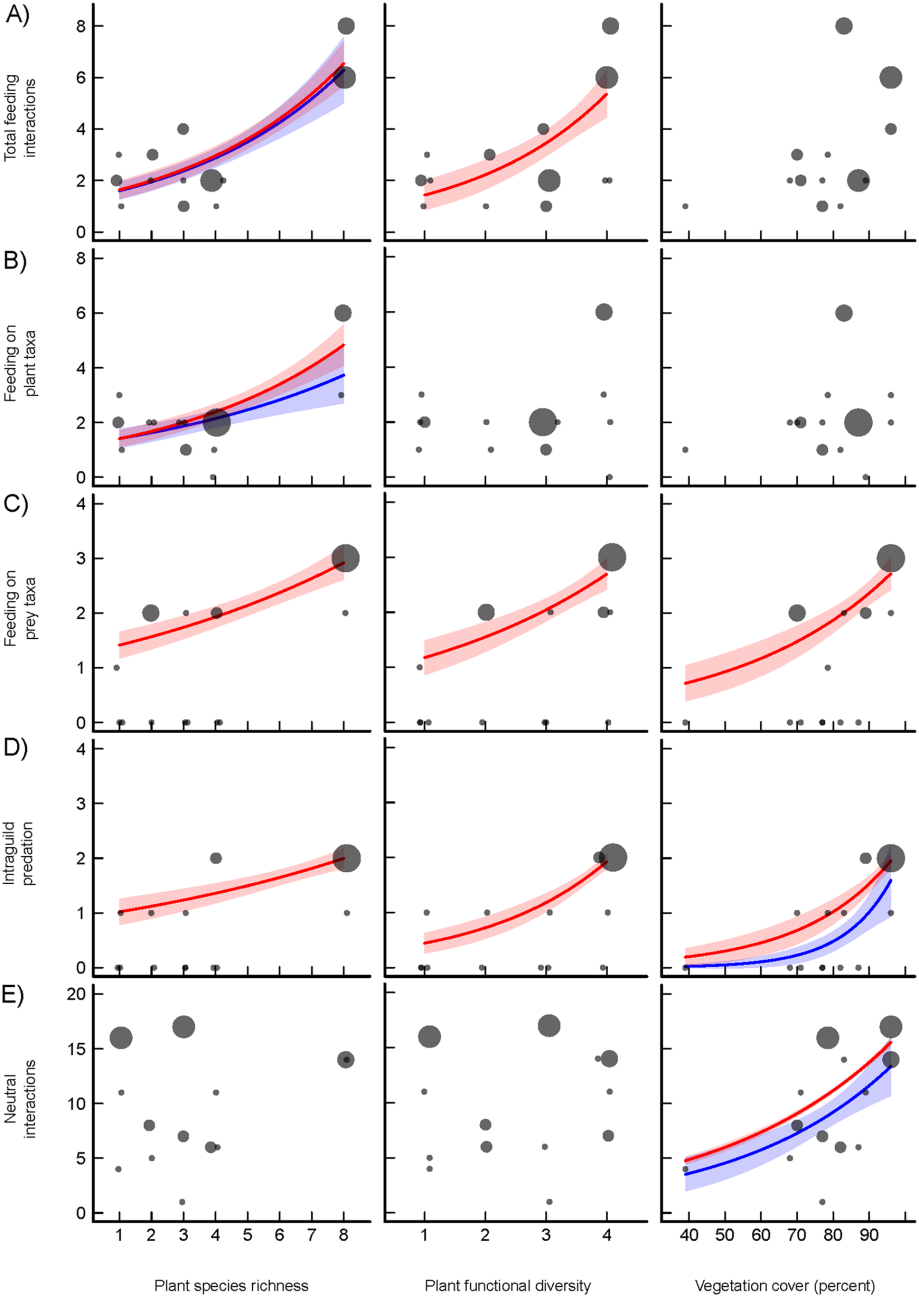
Effects of plant species richness, plant functional diversity, and percentage vegetation cover on feeding interactions and neutral interactions detected in regurgitates of *P*. *melanarius*. Points represent individual plots and are scaled based on the logarithm of the number of sequences, blue lines show GLM predictions, blue polygons show 95% confidence intervals for effects with p<0.05, red lines and red polygons refer to GLMs weighted by the number of sequences. A) Total number of feeding interactions including prey and plant taxa, B) feeding interactions involving plant taxa, C) feeding interactions involving total prey taxa, D) feeding interactions involving intraguild predation, and E) neutral interactions.

Plant diversity affected the total number of feeding interactions and the taxon richness in all food groups including plant-derived food, animal total prey and intraguild prey (Table 1; Fig 2A–2D): the total number of feeding interactions was significantly positively affected by plant species richness and positively but not significantly by functional diversity and vegetation cover. The number of plant taxa detected in the regurgitates increased with the number of sown plant species in the plot. The total number of total prey species increased with plant species richness and vegetation cover, intraguild predation was only affected by vegetation cover. The occurrence of parasitic and phoretic interactions was not significantly related to any of the explanatory variables (Table 2). The richness of neutral interactions was not affected by plant species richness or functional diversity, but increased with percentage vegetation cover (Table 2; Fig 2E). In weighted models, all effects from unweighted models remained significant. Additionally, marginal effects became significant.

**Table 2:** Summary of generalized linear model results on the effect of plant species richness, plant functional diversity and percent vegetation cover on the number of OTUs detected in each interaction group.

Interaction group	Parameter	Estimate	SE	Z-value	P-value
**1a) Total feeding interactions**	(Intercept)	0.275	0.250	1.10	0.295
	Plant species richness	0.195	0.048	4.114	**0.002**
	(Intercept)	0.254	0.457	0.555	0.590
	Plant functional diversity	0.289	0.145	1.996	0.071
	(Intercept)	-1.206	1.298	-0.929	0.373
	Vegetation cover [%]	0.028	0.016	1.797	0.100
**1b) Feeding on plant taxa**	(Intercept)	0.209	0.296	0.708	0.494
	Plant species richness	0.138	0.060	2.295	**0.042**
	(Intercept)	0.435	0.460	0.946	0.365
	Plant functional diversity	0.113	0.155	0.727	0.483
	(Intercept)	-0.102	1.201	-0.085	0.934
	Vegetation cover [%]	0.011	0.015	0.711	0.492
**1c) Feeding on prey taxa**	(Intercept)	-1.087	0.663	-1.639	0.129
	Plant species richness	0.245	0.119	2.067	0.063
	(Intercept)	-1.681	1.041	-1.616	0.134
	Plant functional diversity	0.549	0.306	1.796	0.100
	(Intercept)	-5.989	3.045	-1.967	0.075
	Vegetation cover [%]	0.071	0.035	2.040	0.066
**1d) Intraguild predation**	(Intercept)	-1.379	0.681	-2.023	0.068
	Plant species richness	0.222	0.125	1.769	0.105
	(Intercept)	-2.087	1.076	-1.939	0.079
	Plant functional diversity	0.549	0.316	1.736	0.110
	(Intercept)	-6.728	2.899	-2.320	0.041
	Vegetation cover [%]	0.075	0.033	2.266	**0.045**
**1e) Parasitism**	(Intercept)	-1.099	0.699	-1.571	0.144
	Plant species richness	0.041	0.163	0.252	0.806
	(Intercept)	-1.063	0.902	-1.178	0.264
	Plant functional diversity	0.042	0.315	0.133	0.897
	(Intercept)	-2.562	2.607	-0.983	0.347
	Vegetation cover [%]	0.020	0.032	0.636	0.538
**1f) Phoretic interaction**	(Intercept)	-0.153	0.233	-0.656	0.525
	Plant species richness	-0.004	0.058	-0.073	0.943
	(Intercept)	0.122	0.282	0.434	0.672
	Plant functional diversity	-0.118	0.106	-1.114	0.289
	(Intercept)	0.171	0.682	0.250	0.807
	Vegetation cover [%]	-0.004	0.009	-0.501	0.626
**1g) Neutral interaction**	(Intercept)	2.013	0.270	7.456	<0.001
	Plant species richness	0.059	0.065	0.911	0.362
	(Intercept)	2.008	0.355	5.650	<0.001
	Plant functional diversity	0.082	0.125	0.662	0.508
	(Intercept)	0.345	0.820	0.421	0.674
	Vegetation cover [%]	0.023	0.010	2.325	**0.020**
**2a) Total feeding interactions (weighted)**	(Intercept)	0.296	0.251	1.179	0.263
	Plant species richness	0.198	0.039	5.021	**0.000**
	(Intercept)	-0.080	0.555	-0.145	0.888
	Plant functional diversity	0.440	0.160	2.745	**0.019**
	(Intercept)	-1.084	1.680	-0.645	0.532
	Vegetation cover [%]	0.028	0.019	1.458	0.173
**2b) Feeding on plant taxa (weighted)**	(Intercept)	0.166	0.254	0.653	0.527
	Plant species richness	0.176	0.043	4.134	**0.002**
	(Intercept)	0.015	0.552	0.028	0.978
	Plant functional diversity	0.330	0.168	1.969	0.075
	(Intercept)	0.227	1.875	0.121	0.906
	Vegetation cover [%]	0.009	0.022	0.424	0.679
**2c) Feeding on prey taxa (weighted)**	(Intercept)	0.242	0.200	1.212	0.251
	Plant species richness	0.104	0.031	3.348	**0.007**
	(Intercept)	-0.112	0.362	-0.311	0.762
	Plant functional diversity	0.276	0.101	2.731	**0.020**
	(Intercept)	-1.250	0.798	-1.566	0.146
	Vegetation cover [%]	0.023	0.009	2.642	**0.023**
**2d) Intraguild predation (weighted)**	(Intercept)	-0.076	0.270	-0.280	0.785
	Plant species richness	0.096	0.038	2.529	**0.028**
	(Intercept)	-1.300	0.558	-2.330	0.040
	Plant functional diversity	0.489	0.143	3.416	**0.006**
	(Intercept)	-3.218	1.407	-2.287	0.043
	Vegetation cover [%]	0.041	0.015	2.706	**0.020**
**2e) Parasitism (weighted)**	(Intercept)	-0.006	0.169	-0.034	0.973
	Plant species richness	-0.007	0.053	-0.123	0.905
	(Intercept)	-0.191	0.335	-0.570	0.580
	Plant functional diversity	0.057	0.114	0.500	0.627
	(Intercept)	-0.126	0.686	-0.183	0.858
	Vegetation cover [%]	0.001	0.009	0.146	0.886
**2f) Phoretic interactions (weighted)**	(Intercept)	0.001	0.033	0.026	0.980
	Plant species richness	-0.002	0.012	-0.142	0.890
	(Intercept)	0.011	0.040	0.274	0.789
	Plant functional diversity	-0.008	0.021	-0.391	0.703
	(Intercept)	0.027	0.139	0.198	0.847
	Vegetation cover [%]	0.000	0.002	-0.223	0.828
**2g) Neutral interactions (weighted)**	(Intercept)	2.466	0.040	61.912	<2e-16
	Plant species richness	0.011	0.009	1.178	0.239
	(Intercept)	2.478	0.055	45.236	<2e-16
	Plant functional diversity	0.010	0.019	0.523	0.601
	(Intercept)	0.753	0.128	5.888	0.000
	Vegetation cover [%]	0.021	0.001	14.068	**<2e-16**

All OTUs were assigned to interaction groups (see methods). We tested the effects of three explanatory variables on all interaction groups and compared two types of models. Models 1a-g were based on counts of interactions per plot, while models 2a-g additionally included a weights argument for the number of sequences. All models used 2 degrees of freedom and had 11 residual degrees of freedom. A quasipoisson distribution was used for all models except neutral interactions, for which negative binomial models were fitted. SE = standard error. P-values <0.05 are reported in bold numbers.

**Fig 3:**
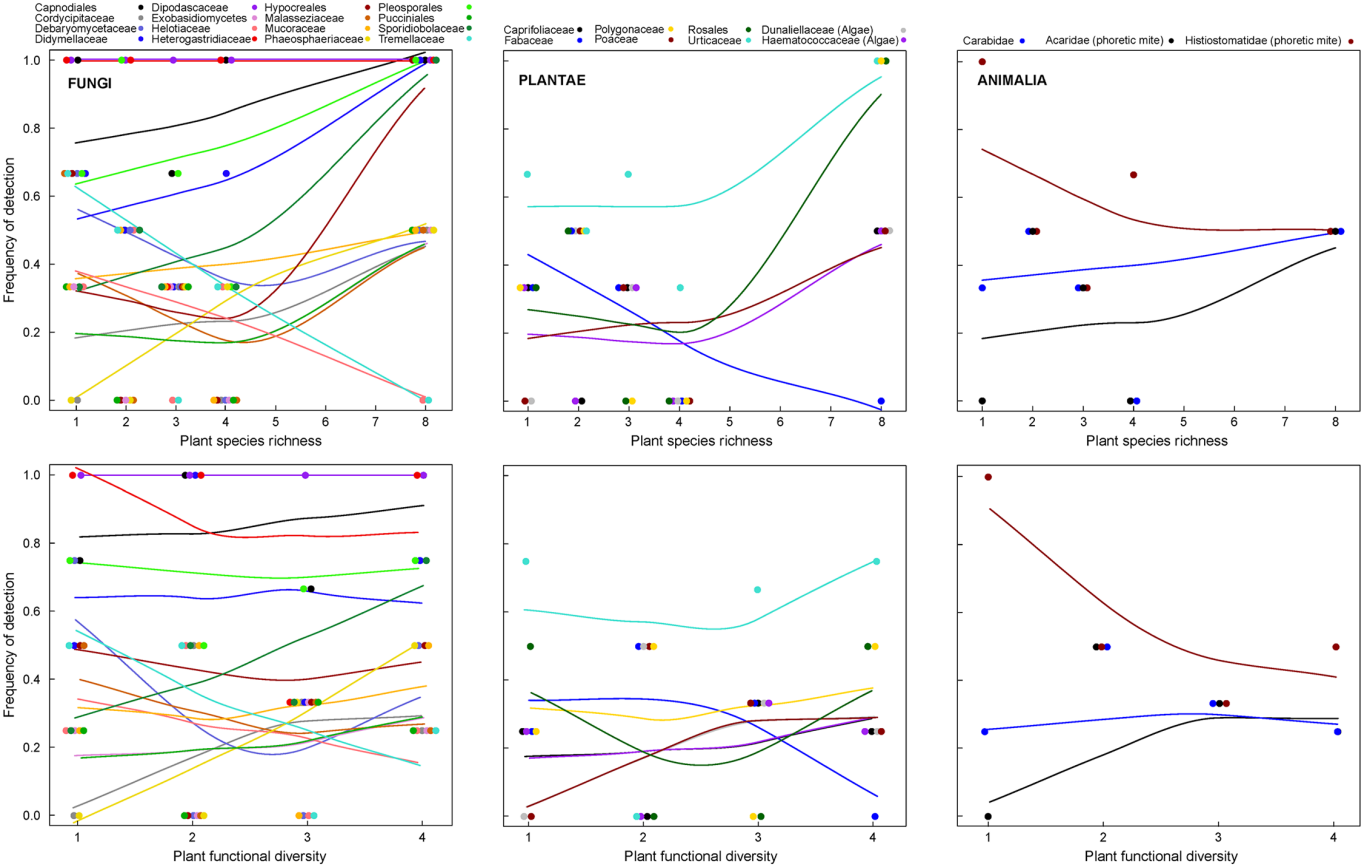
Effects of plant species richness and plant functional diversity on detection frequency of abundant OTUs detected in regurgitates of *P*. *melanarius*. The six panels show the three kingdoms (Plantae, Animalia, and Fungi). Points represent OTUs, aggregated at family level, that were detected in at least two levels of plant species richness. Lines (smoother span = 1.6) show least-squares fits for illustrative purposes only.

Since the identity of OTUs was ignored in the aggregated data analysis, we show in Fig 3 how abundant individual families from the three kingdoms Animalia, Plantae, and Fungi respond to plant species richness and plant functional diversity.

## Discussion

### Assessment and interpretation of trophic and non-trophic interactions

NGS of regurgitates of the omnivore *P*. *melanarius* with primers targeting a spectrum of organisms as broad as eukaryotes allowed us to directly assess trophic and non-trophic interactions involving a wide range of taxa. Any sequencing-based list of interactions will require further validation, as the quality of reference libraries or databases may affect assignment of sequences to taxa. As our study was performed within the framework of a larger biodiversity experiment, we had considerable knowledge on the presence of taxa in the study area, providing extensive species inventories that we used to validate the results. Additionally, for well-studied species such as *P*. *melanarius*, feeding interactions identified by NGS were compared to a broad body of literature on dietary range, feeding preferences, and behaviour. Literature research may also help to reveal which live stage of an animal or type of plant tissue has likely been consumed, as this information cannot be provided by DNA-based food detection. For example seeds are a putative source of plant DNA since they are frequently consumed by *P*. *melanarius* [56] and more often found in guts of the carabid subfamily Harpalinae than pollen or other plant tissue [30].

**Fig 4:**
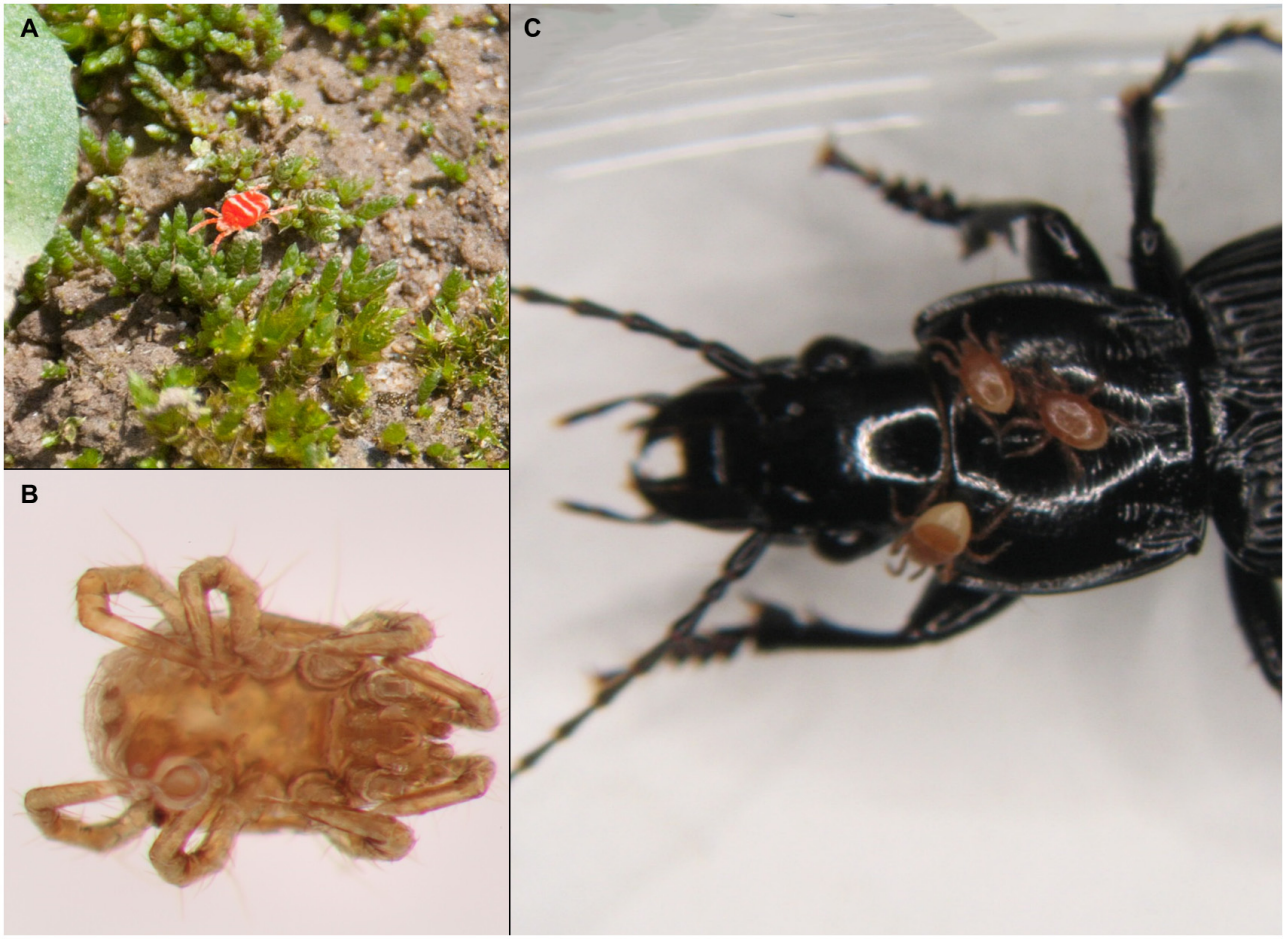
Mites as prey and parasites of *P*. *melanarius*. (A) Predatory mite (Trombidiformes: Trombiculidae) in a plot of the Jena-Experiment. (B) Mite isolated from a gut of *P*. *melanarius* (C). Phoretic mites (Mesostigmata: Parasitidae) on *P*. *melanarius*. Photographs by C. Scherber.

Many taxa we detected are well-known prey of *P*. *melanarius*, including slugs [57,58], earthworms [59], spiders [15], and small beetles [15,60]. More surprising was the detection of grasshopper DNA. Grasshoppers were abundant at the field site during our study (see also [61]), and although it is unlikely that the beetle captured an adult grasshopper, predation on egg pods [62] or scavenging on dead specimen can be considered a likely source of DNA in the gut [63]. Most surprising was the frequent detection of mite DNA, an observation that was supported by mite remains in dissected guts of *P*. *melanarius* specimens collected from the Jena-Experiment (Fig 4A and 4B). Mites are within the food range of ground beetles [29] but their role in the diet of *P*. *melanarius* remains unclear. Generally, the broad dietary range of *P*. *melanarius* reported in the literature [29,30] is well reflected by our NGS-based results on trophic interactions.

Among the non-trophic interactions revealed in the current study, parasitic interactions included an entomopathogenic fungus and a group of parasitic protists. Both could either have been parasites of *P*. *melanarius* or associated with its prey [37]. Despite this uncertainty, detecting parasite DNA in regurgitates of *P*. *melanarius* proves that the beetles were likely exposed to potential antagonists. Mite DNA detected using NGS mayalso indicate phoretic interactions, that is, mites may have used beetles as transporters between habitats [64]. Mite DNA could either originate from mites or their remains, like exuviae, that were externally attached to prey or to *P*. *melanarius* itself and have fallen off during sampling. Additional observations showed that *P*. *melanarius* specimens are frequently infested by mites (Fig 4C).

Most taxa we detected could not be assigned to a specific type of interaction with *P*. *melanarius* and were assumed to be neutral interactions with organisms that coexist with the beetles without affecting them in a particular way. By this simplification, we may have included organisms with a more specific but up to date unknown interaction with *P*. *melanarius*, e.g. yeasts that are beneficial to digestion processes, since the microbiome of ground beetles is largely unexplored [65,66]. Most of the organisms classified as neutral interaction partners could, however, be identified as phytopathogens or saprotrophs for which an effect on *P*. *melanarius* is unlikely. *Vice versa*, the beetle could have contributed to the dispersal of spores [67] but information on the taxon-specific survival through the gut passage is required for assumptions on more specific interactions. It is likely that carabid beetles accidentally ingest all kinds of organisms during feeding or simply by dwelling in their environment, because even non-nutritional material, such as sand, has commonly been reported in their gut contents [30]. Boyer et al. [68] suggest the use of faeces as ‘biodiversity capsules’ for species inventories of the foraging area. Similarly, species composition in regurgitates may provide information on species diversity and ecosystem processes in the beetles‘ habitat.

Further studies are essential to supplement the list of interaction partners by expanding the analysis to bacteria. Facultative bacterial symbionts have an impact on seed consumption by the omnivorous ground beetle *Harpalus rufipes* [65] and may also alter the food choice of field populations of ground beetles.

### Regurgitates as source material for NGS

Our study is among the first to use NGS for the analysis of regurgitates to assess species interactions. Even without the use of blocking primers, only two samples yielded consumer DNA sequences and in one of them there was too much consumer DNA so that the sample had to be removed from the analysis. These results demonstrate that regurgitates contain only little consumer tissue and are asuitable source material for diet analysis of omnivorous or predatory insects because they can be analysed without blocking primers, avoiding drawbacks related to this approach [36,37]. In addition, the DNA recovered from food remains regurgitated from the foregut is likely more intact than from posterior gut sections or faeces. This allowed us to use primers that target a relative large DNA fragment of about 600 bp, which is beyond the recommended size of DNA fragments for molecular gut content analysis (but see [69]), and to assign most sequences to genus or family level. Targeting long DNA sequences may also reduce the chance to detect degraded DNA from prey guts (secondary predation; [70]), or environmental sources. In the present study, we further avoided an overestimation of feeding events by discarding OTUs with low reads.

Defensive regurgitation is not only common in Carabidae [33,71] but also in other coleopteran families commonly occurring in a wide range of ecosystems, for example, Chrysomelidae [72,73], Staphylinidae (personal observation) and Silphidae [74], but also in Orthoptera [75] as well as the larval stages of some Lepidoptera [76]. As regurgitate-sampling is non-invasive it could even be used to analyse the diet of endangered species or gut content samples of an individual at multiple time points. Using regurgitates for NGS based analysis represents a straightforward method to assess trophic and non-trophic interactions. Over all, our results demonstrate that regurgitates are a suitable source material for diet analysis of omnivorous or predatory insects with NGS.

### Effects of plant biodiversity and vegetation cover on species interactions

We conducted our study within the framework of a biodiversity experiment, where aspects of plant taxonomic and functional diversity are experimentally manipulated [39] to allow testing for the effects of plant diversity *per se* on trophic and non-trophic interactions, as opposed to observational studies [77,78]. So far, research on plant diversity effects on higher trophic levels rarely goes beyond measuring species richness and abundance. Although our findings are limited by the small sample size, our study provides insights into how plant diversity affects how well species in a community are connected with each other.

Our results indicate that experimentally manipulated plant diversity may indeed affect interactions between a generalist consumer and its potential food. Both the number of plant and prey taxa detected in regurgitates increased with the number of sown plant species. Plots with high plant species richness support a more diverse consumer community in relation to species poor plots [8] and may provide more potential food items for the omnivorous beetles, thereby facilitating a mixed diet.

Prey detection and intraguild predation also increased with vegetation cover. Large carabid beetles, as *P*. *melanarius* (body size 12–18 mm), prefer structural complexity over open plots because it lowers their vulnerability to predation [79] and may facilitates extensive foraging. The abundance of predators relative to herbivores has been reported to increase with plant diversity [10], potentially increasing the chances that *P*. *melanarius* captures other predators. Hunter [80] suggests that omnivorous consumers preferentially feed on other higher order consumers because they are rich in nitrogen.

In regurgitates of beetles from plots with dense vegetation, we detected more neutral interactions with passively consumed organisms. High vegetation cover may provide a more humid microclimate that facilitates fungi and protists [81,82] and therefore increases the likelihood of encounters with ground-dwelling beetles.

It should be made clear, however, that more replicates and a greater range of consumer taxa will be needed to further elucidate the trends reported here. Nevertheless, our findings agree well with a large body of empirical work [6,8,83] showing a facilitating effect of plant diversity on trophic interactions. Thus, our study presents the intriguing possibility that our understanding of multitrophic food webs can be considerably advanced using molecular tools such as NGS.

NGS-based gut content analysis was so far mainly used to describe the dietary spectrum of species [27,37,84] but is underexploited in research on biodiversity and ecosystem functioning and has rarely been applied in plant diversity experiments. Expanding the spectrum of applications of NGS to address questions and to empirically test theories in biodiversity research is the way forward. With profound knowledge of the species pool and the often extensive data on ecological parameters available in biodiversity experiments, NGS-based gut content analysis can contribute to a mechanistic understanding of diversity effects. Applying very general primers allows assessing trophic interactions on various food types and non-trophic interactions simultaneously in one approach. By using regurgitates as source material, blocking primers for consumer DNA are no longer required and NGS becomes easily applicable even for predators or omnivores.

## Supporting Information

S1 Data OTU table for analysis with S1 R-script. (TXT)

S2 Data Plot information for analysis with S1 R-script. (TXT)

S1 R-Script R script used for statistical analyses. (R)

S1 Table List of removed OTUs. (XLSX)
